# Restoring Connectedness in and to Nature: Three Nordic Examples of Recontextualizing Family Therapy to the Outdoors

**DOI:** 10.3389/fpsyg.2022.768614

**Published:** 2022-03-14

**Authors:** Markus Mattsson, Carina Ribe Fernee, Kanerva Lehmuslaakso, Pekka Lyytinen

**Affiliations:** ^1^The Foundation for the Rehabilitation of Children and Young People, The Mannerheim League for Child Welfare, Turku, Finland; ^2^Department of Child and Adolescent Mental Health, Sørlandet Hospital HE, Kristiansand, Norway; ^3^Faculty of Health and Sport Sciences, University of Agder, Kristiansand, Norway; ^4^Department of Education, Faculty of Education, University of Turku, Turku, Finland

**Keywords:** outdoor therapy, nature connectedness, adventure therapy, family rehabilitation, nature-based family therapy, mentalization, attuned interaction

## Abstract

Mentalization-based family therapy and family rehabilitation represent a rich variety of approaches for assisting families with difficult interaction patterns. On the other hand, adventure therapy methods have been successfully used with families to offer them empowering experiences of succeeding together against difficult odds and to improve communication between family members. Further, the health promoting qualities of spending time outdoors are now well established and recognized. The Nordic approach to mentalization-based family rehabilitation combines adventure, outdoor, and systemic therapy. We provide three examples of nature-based family rehabilitation practices that are delivered as brief, multi-family psychological interventions taking place in nearby nature and aiming to support sustainable, systemic change. The current contribution is a description of clinical practice, not a systematic review or a formal evaluation. We propose that recontextualizing mentalization-based family rehabilitation to the outdoors can not only provide added health benefits, but also strengthen intra-familial attuned interaction and emotional connectedness. The outdoor adventure provides the families with embodied, multisensory experiences of verbal and, especially, non-verbal interaction that can be usefully examined through the lens of theory of mentalization. The concreteness of adventure experiences is particularly beneficial for families that have difficulties in verbal communication and/or utilizing executive functions, perhaps due to neuropsychiatric traits, intellectual disabilities, or learning difficulties. Furthermore, outdoor adventure can support the participants’ connectedness to nature.

## Introduction

Humans are social beings and have a fundamental need to belong (Fiske, 2018). We thrive when feeling connected to other living things—people, animals, and nature—but also to more abstract things such as a hopeful future and meaningful values (Baumeister and Leary, 1995). Among these connections, the parent-child relationship is particularly salient for the emotional, social, and mental wellbeing of both the parent and the child, and the attachment to our primary caregivers affects our psychosocial development from infancy into adulthood (Siegel, 2012).

Numerous factors on personal, interpersonal, and societal levels influence intra-familial relations. Mental health problems, substance abuse, parental feelings of incompetence, and various forms of preoccupations and stress typically limit positive parent-child interactions and may hinder supportive parenting efforts (Bode et al., 2016). Certain traits of children, such as Autism Spectrum Disorder (ASD) and Attention Deficit Hyperactivity Disorder (ADHD), are associated with parental stress, lack of self-efficacy, marital problems, and impact on siblings, which then potentially feeds back to behavioral problems of these children (Karst and Van Hecke, 2012). Under these circumstances, it becomes challenging for parents to maintain attuned connection and empathize with their children, while teaching them social skills (Milton, 2012; Dugdale et al., 2021).

Family therapy (Carr, 2019a), and systemic approaches in general, have been shown to be effective treatment modes for various problems originating with the child, the adult (Carr, 2019b), and the dynamics of the family system. The jury is still out when it comes to the relative effectiveness of family therapy as compared with individual therapy (Carr, 2019a and the research cited therein), even though this depends on the presenting issue and the kind of family therapy employed. Specifically, behavioral parent training (BPT) that focuses on teaching the parents concrete strategies for directing and controlling the behavior of children (e.g., using praise and discipline) has been convincingly shown to be effective in families with children with neurodevelopmental disorders such as ADHD (Daley et al., 2018) or ASD (Postorino et al., 2017). BPT has also been shown to be more effective than individual therapy (McCart et al., 2006), and involving the father in the treatment appears to increase its effectiveness (Carr, 2019a).

Further, in families with children with ADHD, integrated parent-youth interventions have been shown to reduce family stress in addition to positively affecting parenting behaviors, while treatment targeted at youth with adjunctive parent involvement may positively affect relationship quality within the families (Babinski and Sibley, 2021). Family-based interventions targeting mood disorders, such as childhood anxiety and depression, are more effective than individual treatment when considering long-term outcomes (Carr, 2019a). This may especially be the case when it comes to children with ASD and childhood anxiety (Goger and Weersing, 2022). Family therapy offered in a multi-family format includes the added group elements of mutual self-help and peer support built into the therapeutic approach (Jewell and Lemmens, 2018). There is evidence that multiple family therapy can be an effective intervention across a range of conditions, including mood disorders, even though its effectiveness for childhood ADHD and ASD is still anecdotal and studies of higher quality have been called for (Cook-Darzens et al., 2018; Gelin et al., 2018).

Human health and wellbeing also depend on the relationship between humans and the natural world (Hartig et al., 2014; McMahan and Estes, 2015), conceptualized sometimes as nature relatedness (Nisbet et al., 2009), inclusion of nature in self (Schultz, 2002), emotional affinity toward nature (Kals et al., 1999), and nature connectedness (Mayer and Frantz, 2004; Capaldi et al., 2014). Some of the concepts emphasize cognitive appreciation of nature, others emotional attachment, and our material dependence on nature (Petersen et al., 2019). According to the biophilia hypothesis, there exists a specialized biological mechanism that supports our innate attraction to nature (Wilson, 1984). Finally, the idea of human-nature kinship (Harper and Doherty, 2020) is nothing new; rather, it has remained at the core of traditional cultures and their mental health practices across centuries; see, e.g., Watts (1961) on eastern and western psychotherapeutic practices.

Indeed, time spent outdoors is known to be beneficial for us in many ways, leading to lowered stress levels, restored attentional capacities (James et al., 2015), and improved mood (Cooley et al., 2020). We use the term “main effects of nature” to refer to the general health benefits of nature contact. Natural environments are considered to be particularly beneficial for children with neuropsychiatric traits such as ADHD and ASD (Kuo and Taylor, 2004; Faber Taylor and Kuo, 2011; Di Carmine and Berto, 2020) as well as for families in general. Spending time together outdoors appears to enhance communication, which becomes more responsive and connected when compared to an indoor setting (Cameron-Faulkner et al., 2018).

Still, the ordinary practice of psychotherapy in western cultures is based on reflecting on experiences and verbal processing indoors (Cooley et al., 2020). Yet several researchers have argued that talking is not sufficient and that embodied experiences are needed as the basis of reflection (Krueger, 1989; Fonagy et al., 2002; Kristmannsdottir, 2012). Natural environments provide ample opportunities for reflection and bodily activation, and a recent meta-synthesis on therapeutic work in outdoor spaces describes several preconditions and potential mechanisms for this to happen (Cooley et al., 2020). First, both the client and therapist need to feel comfortable in the chosen outdoor setting. The different approaches, ranging from walk-and-talk sessions in urban parks to expeditions in remote wilderness, involve different levels of interaction with nature. The role of nature can similarly vary from a passive backdrop to an active participant in therapy, offering metaphors and opportunities to analyze the behavior of the client in real time. Further, the outdoor environment facilitates the formation of therapeutic alliance and provides freedom to express different emotions, together with the freedom from the role of being a patient. Interestingly, the natural environment also provides opportunities for the patients to integrate what is going on in their minds and bodies and for the therapists to tune in to the rhythm of the client (e.g., experiencing physical empathy through walking together at the same pace).

In addition to building on previous systemic therapeutic approaches and recognizing the effects that nature has on our physiological and mental wellbeing, and on the relationship between the therapist and the client, our work builds on previous family-therapeutic work performed in natural settings. It was argued already by Clark and Kempler (1973) that the results of family therapy carried out in an office environment do not always generalize to the everyday life of the family. On the other hand, working outdoors in an environment that resembles the home environment while still differing from it in certain key respects offers certain important benefits. For instance, taking a break from everyday life as a family and being together in a novel natural setting enables the families to receive feedback from multiple sources (each other, the therapists, and other families) and the opportunity to try out the new insights immediately with the support of the group and therapist(s) as needed. In traditional family therapy, temporal gaps between receiving a new insight and getting to try it out in practice may dilute the effectiveness of the therapy. A more recent discussion on multi-family adventure therapy (Swank and Daire, 2010) echoes these sentiments, while mentioning additional benefits of the multiple family format, such as the decreased feeling of isolation, which in itself leads to improvement in within-family dynamics. Similarly, they argue that receiving constructive feedback from other families combined with observing how the other families interact may also underlie positive changes. Further, outdoor adventure engages the whole body, providing a holistic experience in an environment that both challenges and supports family dynamics and making it almost a necessity to help and interact with others. Finally, sharing the outdoor environment with others provides immediate feedback to the participants on their own actions in a way that can be examined later on with the program staff.

Qualitative studies make similar observations time and again, whether the context is family recreation, outdoor learning, or outdoor therapy: Participating in adventure activities in natural settings is argued to be beneficial for families due to the novel environment assisting the families in becoming aware of previously undiscovered familial resources, strengthening emotional bonds between family members, and enhancing communication between family members (Freeman and Zabriskie, 2002; Huff et al., 2003; Overholt, 2019). Some mechanisms of change may involve improved listening skills among the family members, the practice of taking “time-outs” from heated discussions, and learning to communicate in an assertive manner when required (Liermann and Norton, 2016).

Family-therapeutic outdoor therapy has still built on different theoretical ideas. Bandoroff and Scherer (1994) demonstrated ways of integrating techniques of structural family therapy into the practice of wilderness therapy. More specifically, they advocated a systemic understanding of change processes and argued that insights gathered as a family are more likely to transfer to everyday life compared to beneficial effects that are experienced by the youth when they participate in individual wilderness therapy. Family-directed structural therapy, on the other hand, is an integrative approach to family therapy that builds on the ideas of the strength-focused approach, where the families themselves are made responsible for the change process. Applying this approach to a therapeutic family camp, McLendon et al. (2009) found it to be an effective intervention for a variety of core presenting issues, while simultaneously also improving family cohesion and adjusting family roles.

While the structural therapies focus on the roles occupied by the family members, adventure-based counseling has also been described from an Adlerian perspective, which emphasizes the children’s need to belong while at the same time noting their goal of establishing personal identities separate from their families (Christian et al., 2017). As these needs sometimes lead to adolescents behaving in ways not condoned by their parents, the role of the counselor becomes to enable the parents to perceive the needs that may underlie the behavior of the adolescents. Further, DeMille and Montgomery (2016) illustrate how narrative family therapy and storytelling can be applied in outdoor therapy to create mutual understanding between adolescents and their parents. Finally, Norton et al. (2019) describe ways of incorporating the principles of trauma-informed care into multi-family adventure therapy and describe its effects such as successfully decreasing trauma symptoms of children, most notably depression and anxiety, and improving communication within the families.

While still limited, outdoor adventure has also been applied in dyadic work with families. Overholt (2019) describes an intervention aimed at supporting fathers in a society where the expectations related to parenting are changing and fathers are expected to participate in childcare more than previously. Overholt (2019) used identity theory to examine changes in father-child relationships, reporting that the main outcomes included enhanced trust, communication, and mutual reliance among the fathers and children. While Overholt (2019) emphasized the role of wilderness, she also speculated whether less remote nature experiences may promote the relationship between parent and child to the same extent. Adopting a slightly different gendered perspective, Izenstark et al. (2021) have demonstrated the benefits that simply walking together outdoors may have on interpersonal affective dynamics and communication within mother-daughter dyads.

Outdoor family therapy has also been specifically targeted at families with children with ADHD or ASD. Greenfield and Senecal (1995) explored recreational multi-family therapy for families with children with ADHD, anxiety, or developmental disorders. They found that the adults learnt new parenting skills and that recreational outdoor activities were helpful in repairing family conflicts. In addition, the group format diminished the parents’ sense of isolation, shame, and embarrassment. Family-centered nature-based therapy has also been noted to improve interactions between parents and children with ASD (Ramshini et al., 2018). In a similar vein, participation in a family leisure camp for children with ASD initially increased different aspects of family functioning, even though most of the effects no longer differed from baseline at 6-months follow-up (Wenzel et al., 2020).

When assessing the relevance of the work cited above to the approach described in this contribution, it is necessary to note that much of this work has been carried out either in the North American or the Australian cultural sphere, which differs in important ways from the Nordic outdoor culture. The *friluftsterapi* approach (Gabrielsen and Fernee, 2014) is emblematic of the Norwegian *friluftsliv* way of life (Fernee et al., 2015) and bears important similarities to the approach described below. Defining characteristics of the friluftsterapi approach—that also set it apart from the view of nature and coercive practices occurring in US wilderness therapy (Dobud, 2021; Harper et al., 2021)—include the following: (1) the role of nature and the relationship between the participants and nature occupy a central role, being in important respects defined by the ancient Nordic behavioral code of freedom to roam (Norwegian: *allemannsretten*; Finnish: *jokamiehenoikeudet*), (2) the interventions are (relatively) brief and carried out (relatively) close to home so that the potential changes can more easily be incorporated into the daily lives of the participants, (3) the participants are involved as active actors in all decisions that concern them—including, importantly, whether to participate or not, (4) self-realization as a goal of the intervention is understood as a relational concept, referring to the individuals identifying themselves with their surroundings in nature (Næss, 1990, p. 8–9) and as a part of the group in which they belong, and (5) the interventions are grounded in the Nordic model of universal healthcare for all, wherein the economic resources of the participants do not determine who has the possibility to participate.

In this paper, we provide Nordic examples of relocating multi-family interventions from indoor clinical settings to nature, focusing, in particular, on how such recontextualization may augment intra-familial connectedness. We describe three versions of nature-based rehabilitation where attuned interaction between parents, caregivers or grandparents, and children is facilitated through mentalization-based outdoor adventure. We describe similarities and differences between mentalization-based work taking place indoors and outdoors and conclude that relocating our interventions to the outdoors offers distinct benefits. We also briefly describe the assumed causal pathways underlying some of these effects.

## Nature-Based Family Rehabilitation: Three Nordic Examples

Since 1994 the Foundation for the Rehabilitation of Children and Young People, the Mannerheim League for Child Welfare (here: the “Foundation”) has worked with families with children who have special needs. Nature- and adventure-based methods have been essential through the years when working with family groups and single families. Hundreds of families—most of them with children with neuropsychiatric traits related to ADHD and/or ASD—have attended, and rehabilitation methods have been shaped to meet the needs of this population. The rehabilitation services at the Foundation are characterized by the principles of Need-Adapted Treatment (Alanen, 2009), an integrative approach (Alanen, 2009; Seikkula, 2011) that involves combining different therapeutic methods in a flexible manner. Current research on the effectiveness of psychotherapy methods is closely followed and actively applied in clinical practice. The therapeutic approaches range from psychodynamic to solution-focused and systemic family therapies.

Our work builds on the following ideas: (1) A relational approach (Karvonen et al., 2016) to rehabilitation where an adult-child dyad is the minimum unit of intervention and the family-therapist relationship is in focus, (2) A systemic view of (extended) families (Cox and Paley, 1997), (3) A dialogical approach where the intervention and its aims are planned in collaboration between professionals and clients (Seikkula, 2011), and (4) Mentalization-based work where the aim is to support the dyads in co-regulating emotions in a way that (5) offers the parents experiences of success in supporting their children when drawing upon existing but perhaps underutilized resources (Asen and Fonagy, 2012). In particular, strengthening the parents’ mentalization skills of understanding their children’s experiences (Pajulo et al., 2015; Pyykkönen, 2017) is an efficient way of influencing their interaction patterns and creating both flexibility and stability in relationships (Slade, 2005). Our approach entails holistic, person-centered recovery (World Health Organization, 2021), where dialogicity is seen as a way of life, a strong commitment to engage in a dialogical relationship with other people and with nature (Seikkula, 2011). The adventure methods in use at the Foundation are described in Virtanen (2011).

While the principles can be applied indoors, working with Video Interaction Guidance (VIG; Kennedy, 2011) or various activity-based methods, we prefer natural settings for the kinds of reasons referred to as “main effects of nature” in Introduction. Similarly, certain common elements to all adventure/wilderness therapy were described in Introduction.

**Figure 1:**
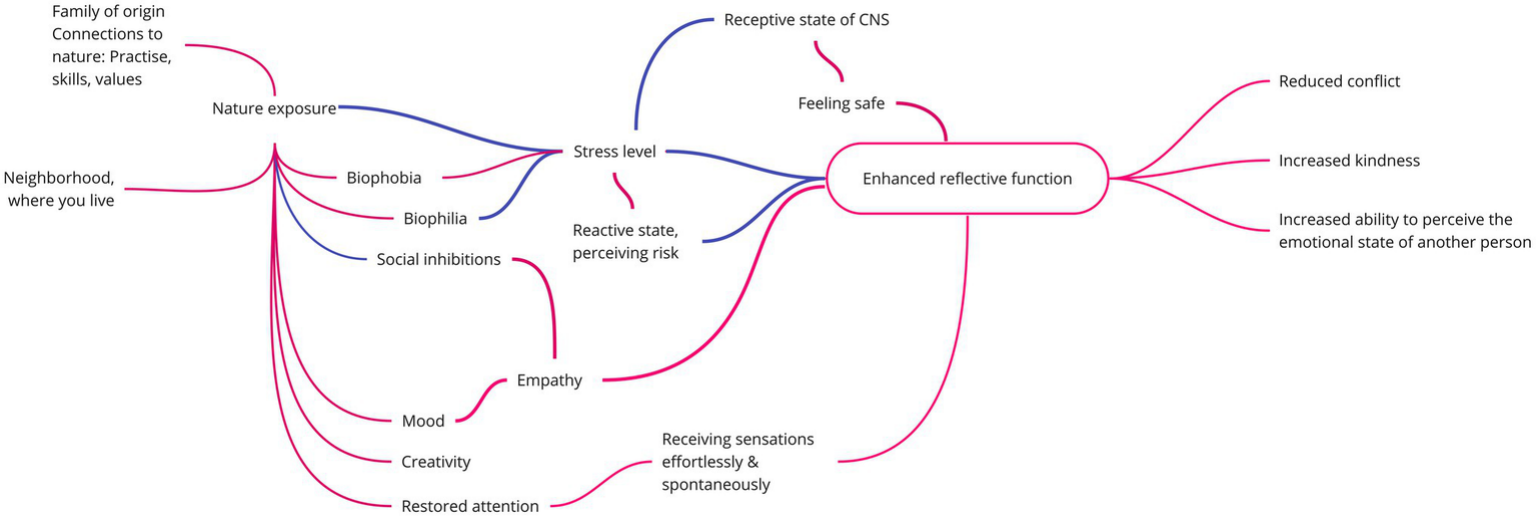
The theoretical framework underlying SMOT. The red lines depict assumed positive relationships, whereas the blue lines illustrate assumed negative ones. CNS, Central nervous system.

The main elements of the systemic, mentalization-based outdoor therapy (SMOT) applied at the Foundation are illustrated in Figure 1.

When combining outdoor family rehabilitation with mentalization-based work, we apply the central ideas put forth by Asen and Fonagy (2012), which are compatible with our relational (Karvonen et al., 2016; Kykyri et al., 2017) and dialogical approach (Anderson and Goolishian, 1992; Seikkula, 2011). These include:

1. Openness to discovery and a genuine interest in others. It is a central goal or aim for us to abstain from making assumptions of what others feel or think. Sometimes we succeed, at other times fail miserably—and try to learn from the experience.
2. Practicing not-knowing the mental state of the other. Even at best, we can make an educated guess of what the other person is feeling, thinking, or going through. Because of this, we try to uphold an atmosphere of safe uncertainty (Mason, 1993): we do our best to keep an open mind when it comes to the mental state of the other, what may be causing it, and what might be the most effective solution to a given challenge.
3. Reflective contemplation and perspective taking. For us, this means not only facilitating the clients’ reflection on their inner experiences, but also a constant reflection on mutually shared activities and interaction.
4. Forgiveness: Even the reactions that are most difficult to understand make sense in the other person’s psychic reality.
5. Impact awareness: Aiming to understand how one’s own thoughts and actions affect others.
6. Humility: All participants, professionals and clients alike, can learn from each other; no one pretends to possess final knowledge regarding what is best for the clients (*cf*. dialogicity).
7. Playfulness and not taking oneself too seriously may catalyze the change process.
8. The belief in changeability links our approach to previous solution-focused work (e.g., Gass and Gillis, 1995), implying profound optimism regarding the clients’ strengths and resources (Berg, 1994). Finally,
9. Assuming responsibility and accepting accountability as principles modeled by the staff and applied by the clients. This is made possible by the professionals’ seemingly passive role during adventure, referred to as “positive indifference” (Virtanen, 2011, p. 67).

There are certain benefits to working outdoors based on these principles. First, the main effects of nature help create the preconditions for mentalizing interactions (Fonagy and Target, 1997) by lowering stress levels and supporting a positive mood state. Second, outdoor adventure provides a safe environment for experiencing (bodily) stress and making it through the situation together. While excessive stress effectively prevents mentalizing (Asen and Fonagy, 2012), we see slight stress as beneficial: when experienced in a novel environment, it prompts the development of new coping mechanisms and offers the families an experience of successfully managing the situation together. Successful mentalizing is possible in the receptive state, but it need not always be the parents who stay in control; it may be empowering—and in line with their developmental task (Erikson, 1950)—for older children to manage a certain situation better than their parents. These considerations regarding stress similarly apply to therapists, as facilitating the supportive conditions mentioned above becomes difficult with rising stress levels.

**Figure 2:**
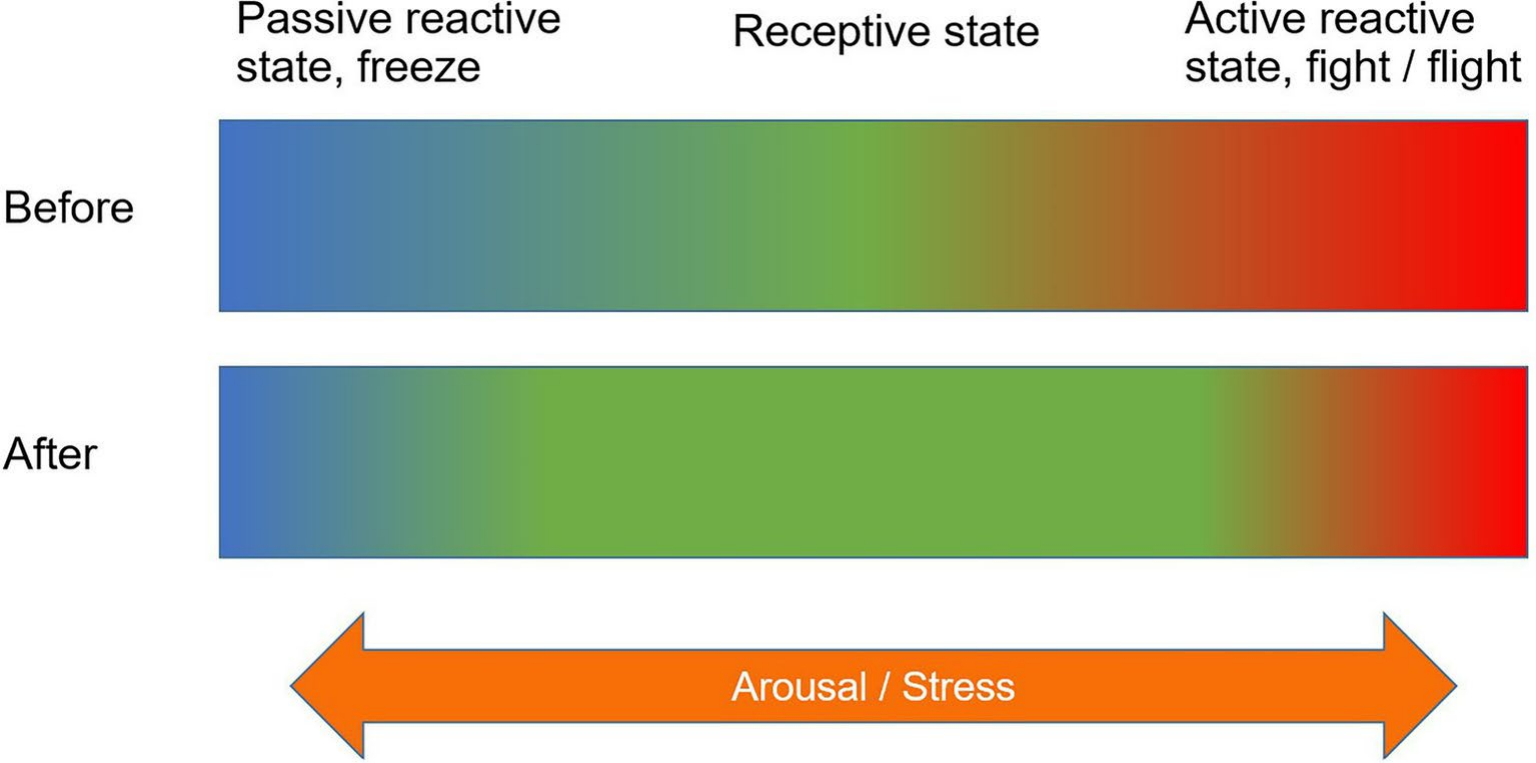
The shared goal of sensitive parenting and SMOT: extending the mental state conducive to mentalization and controlling one’s emotions.

The relationship between stress and mentalization is one of the central factors in SMOT. In Figure 2, the mental state conducive to mentalization is referred to as the receptive state (Siegel, 2012, p. 18.4–18.5). Excessive stress levels will lead to either the sympathetic nervous system (active reactive state) or the parasympathetic nervous system (passive reactive state) becoming overactived (Fonagy and Luyten, 2009; Siegel, 2012, p. 18.4–18.5), both of which make it impossible to relate to others through controlled mentalization (see Figure 1 in Fonagy and Luyten, 2009, for another visualization of the same issue). According to SMOT, the shared goal of sensitive parenting and adventure therapy is to extend the desirable receptive state, and along with that helping children learn how they react and how to regulate their emotions and reactions under stress.

Third, the fact that adventure and subsequent reflection take place in the same environment is beneficial for families with children with neuropsychiatric traits. While executive difficulties can complicate engaging in traditional psychotherapy, the adventure environment may function as a recall cue which facilitates the verbal processing of the experience. Further, outdoor adventure supports the application of the mentalizing loop (Asen and Fonagy, 2012) in real time, when processes related to family dynamics unfold. Guided by the professionals, the family members get a chance to attempt to understand each other’s (bodily) experiences, and to verbalize these, if possible. Finally, the group format provides the families with vicariously experienced examples of well-functioning interaction.

The three developmental projects introduced below are financed by the Funding Centre for Social Welfare and Health Organizations in Finland. The three examples are all low-threshold interventions, meaning that no psychiatric diagnoses are required in order to participate in these nature-based services.

### Fathers and Sons Up in the Trees: Attuned Interaction Through Mentalization-Based Outdoor Adventure

Years of clinical experience at the Foundation have shown that when children have neuropsychiatric traits, fathers face the risk of losing their role as an active caregiver. The fathers may lack social and emotional skills needed to maintain a close contact with a resisting child. In addition, mothers may tend to dominate the caretaking while clinical practices may also feel foreign for the fathers. The parents may have neuropsychiatric traits of their own, the traits being highly heritable (Lichtenstein et al., 2010), which in itself affects their parenting. Cultural influences on masculine roles also affect the fathers’ involvement and attitudes (Singh, 2003).

The developmental phase of the project “Fathers and sons up in the trees” took place during the years of 2015–2017 and the project has since then continued on a yearly basis. The target group is boys between 10 and 16 years of age with neuropsychiatric symptoms and their fathers. The goals include: (1) supporting the developing autonomy, (2) preventing the risk of social exclusion, and (3) promoting age-specific, meaningful leisure activities for the boys.

“Fathers and sons up in the trees” was offered as a group intervention. In the main intervention, the father-son dyads participated in adventure-based activities over three weekends with overnight stay outdoors or indoors. The weekend gatherings consisted of up to six dyads. The dyads chose three of the four main adventure themes such as climbing, canoeing, hiking, and group adventure challenges. The groups were not closed and their composition thus varied between the weekends. In addition to weekend gatherings, the dyads had two opportunities to try a leisure activity of their choosing, based on the interests of the sons.

During the developmental stage (2015–2017), a total of 56 voluntary dyads participated, and evaluative data were collected and analyzed from 55 of these. The key item in the Client Satisfaction Questionnaire was “The participation was beneficial for me.” A total of 64% of the sons (*n* = 50) and 90% of the fathers (*n* = 51) agreed or strongly agreed. In the post-interview, the fathers and sons reported positive changes in their relationship and the quality of their interaction, in addition to enhancement in the sons’ skills in self-regulation of emotions and social conduct (Lyytinen, 2018).

The apparent success of the project is likely to depend on several factors. Intuitive parenting (Parsons et al., 2017) refers to a hard-wired, universal disposition to care for children. The adventure program offers the fathers a possibility for this and strengthens the participants’ attuned interaction (Kennedy, 2011), referring to a harmonious, responsive, and reciprocal relationship. During and after the intensive experience, the dyads may experience moments of secondary intersubjectivity, a process of experiencing and expressing emotions in a dialogue (Trevarthen, 2009; Hughes, 2011). Adventure activities involve a perceived risk (Harper et al., 2019) and strengthen the fathers’ mentalization as they want to protect the sons against the risk that they, too, experience. Finally, the group format offers possibilities for discussing important topics spontaneously with peers.

### Girl You Are a Pearl: Supporting the Relationship Between Mothers and Daughters in Nature

Girl, You Are a Pearl! (GYAP) was a three-year project (2018–2021) dedicated to supporting the relationship between teenage daughters and their mothers, intended for girls with incipient mental health issues. Besides being intrinsically valuable, supporting the mother-daughter relationship aims to reinforce the girls’ mental health as a means of primary prevention.

The project’s format consisted of three group meetings, six individual meetings, and collaboration with school healthcare, social welfare, and specialist medical care. The sessions incorporated mentalization-based methods, such as VIG, art therapeutic activities, and shared nature experiences.

When the dyads were asked to name goals for participating, the three most common answers were: (1) a strengthened emotional connection, (2) improved communication, and (3) increase in time spent together. A total of 90% of respondents considered that participation helped meet their objectives.

GYAP aims to support one of the most important developmental tasks in adolescence: becoming independent (Steinberg, 1990; Coleman, 2011). When parenting a teenager, one must provide space for independence and balance this with setting boundaries and providing support (Steinberg, 2001; Collins and Laursen, 2004; Coleman, 2011). The maternal relationship is of paramount importance in the development of teenage girls’ self-esteem (Näre, 2010). The daughters identify with their mothers, which brings security, but can hinder their development into independent women (Rutanen, 1982). Thus, effective and open communication and emotional connection were essential; in GYAP, these are supported by the use of VIGMLL^®^ counseling. VIG also helped the mothers become more aware of their abilities to build a reciprocal connection with their daughters.

When considering the effects of nature in GYAP, physical activity outdoors and a flow state induced by nature contribute to reducing anxiety (Humberstone, 2013; Korpela et al., 2014; Mao et al., 2020), which is a common symptom for the girls. Time spent outdoors may create transcendental experiences such as feelings of being one with nature, which may lead to the flow state. In addition, group activities are especially suitable for adolescents who have a developmental need to associate with peers (Bandoroff and Scherer, 1994). Peer support was a key element in the project, both for mothers and daughters.

Shared nature experiences and adventure activities provide conditions for demonstrating competence and overcoming self-imposed limitations. Self-image is improved through developing self-efficacy and self-regulation skills (Bandoroff and Scherer, 1994). Mechanisms such as these support the daughters’ mental health and mothers’ view of themselves as capable parents.

### Out Into the Woods With Grandparents and Grandchildren With Special Needs

Grandparenting children with special needs is a new and stressful life situation for the grandparents, with potential for conflict with their own children (Klasén McGrath, 2008). It evokes feelings ranging from pride and joy to mourning lost opportunities, uncertainty over the child’s future, anger over injustice, and social shame related to stigma (Hillman, 2007; Fiske et al., 2014). On the other hand, it may provide a sense of meaningfulness that is invaluable for the grandparents when facing the life tasks of Adulthood II: rediscovering their identity and finding new ways of being useful (Erikson, 1950; Bateson, 2010).

Still, interventions aimed at grandparents are as greatly needed as they are scarce (Hillman, 2007; Fiske et al., 2014; Zakirova-Engstrand et al., 2021). The grandparents need both information and skills related to childcare (Klasén McGrath, 2008; Zakirova-Engstrand et al., 2020). In addition, both them and children on the neuropsychiatric spectrum benefit from actively doing things together (Wright et al., 2012; Pandya, 2020), especially when the focus is on the childrens’ strengths (Wright et al., 2011).

Out into the Woods with Grandparents and Grandchildren (OWGG) builds on these ideas and applies SMOT to offer rehabilitative adventure experiences to grandparent-grandchild dyads or triads in nearby nature for strengthening their mutual relationships and connection with nature. In addition, psychoeducation is offered to the grandparents in a group format (as per Zakirova-Engstrand et al., 2021). The OWGG process consists of two trips to nearby nature in a group of grandparents and grandchildren combined with two discussion group meetings for the grandparents only. The project is currently ongoing (2020–2022).

Preliminary results show that the intervention helps the grandparents understand their grandchildren better, provides them with applicable skills, and increases their compassion toward the grandchildren. We believe there are several underlying causal mechanisms. First, psychoeducation, group discussions, and positive shared experiences may lead to the grandparents viewing the children in a more balanced and positive light than previously. Second, the threshold for attending the discussion group for the grandparents is lowered by first offering them and their grandchildren a shared nature experience in a group format. Further, sharing experiences with grandchildren, and being able to teach and learn, functions as a partial answer to the life tasks facing the grandparents (Bateson, 2010).

The forest environment provides certain distinct benefits. Many grandparents are used to a culture of social shame related to their grandchildren (e.g., noise in public places), but nature is forgiving in this respect, making it easier for the participants to focus on each other. As neuropsychiatric traits are largely heritable (Lichtenstein et al., 2010), the grandparents may well share the properties of their grandchildren. Because of this, we aim at facilitating the discussion groups out in nature, which allows one to move more freely and take breaks as needed. Further, the grandparents function as agents of cultural transmission, helping the children form a relationship with nature. The grandparents get to advise and assist the grandchildren in learning important practical and interpersonal skills, and experiences such as these may become integrated as *vertical polyphonic voices* (Seikkula, 2008) that provide the children with strength and comfort in difficult times later in life—through such experiences, the children always carry their grandparents with them through life.

## Future Directions: Toward Sustainable, Systemic Change

We have argued that mentalization-based adventure therapy offers certain distinct benefits over working indoors, largely because of the concrete, embodied nature of the adventure activities. Our approach can be applied in nearby nature, based on the friluftsliv way (Fernee et al., 2015), according to which outdoor pursuits do not require expensive gear and technical skills. Nearby nature is accessible, making the outdoor practice time- and cost-efficient. It is also easier for the families to maintain similar outdoor pursuits after the intervention, thereby maintaining potential benefits over time, hence achieving sustainable, systemic change.

SMOT shares certain similarities with the approaches related to adventure programming and wilderness therapy described in the introduction. The holistic nature of adventure therapy and the various benefits related to working with groups of families (Swank and Daire, 2010) very much apply to our work as well. The focus on the resources of the families that they perhaps were not aware of to begin with is another common factor between SMOT and the previous work (Freeman and Zabriskie, 2002; Huff et al., 2003; Overholt, 2019). There are also parallels with the Adlerian approach (Christian et al., 2017): it is of central importance to us to always keep the needs of the participants in mind and to use intentional concepts (ones that refer to mental states) as explanations of behavior. On the other hand, SMOT is in quite a stark contrast with the ideas of structural family therapy (Bandoroff and Scherer, 1994): for us, the role of the therapist is much less directive (*cf*. “positive indifference,” Virtanen, 2011, p. 67) and our work is much more based on a dialogue with the participants. In these respects, many of the ideas of trauma-informed adventure therapy put forth by Norton et al. (2019) can be thought of as shared premises with SMOT. Still, the ideas of enactment and therapeutic intensity (Bandoroff and Scherer, 1994) apply to SMOT, as well: the former as is, and the latter as a level of stress that is conducive to mentalization.

The friluftsliv way and a fortiori, the Norwegian friluftsterapi approach (Gabrielsen and Fernee, 2014) are especially important points of reference for SMOT. Similarly to friluftsterapi, our approach is more focused on relationships between participants, and less on individual self-realization in comparison with the US wilderness therapy tradition. Arguably, SMOT, with its explicit focus on mentalization and a systemic point of view toward the life situation of the individual, brings the relational understanding still a step further than the friluftsterapi way. In SMOT, the smallest unit of intervention is a dyad composed of a child and an adult, whereas friluftsterapi interventions are traditionally aimed at groups of individual youth, including parents and social network on the introductory day and the closing seminar only (Fernee et al., 2019).

Certain differences between the US and the Nordic wilderness therapy models were mentioned above (see the numbered list in the Introduction). These differences are related to each other, even though that may not be obvious at first sight. Take the length and location of the intervention, and its relationship with how self-realization is interpreted within the US and the Nordic traditions: in the former, individual patients are removed from their social networks and surroundings—sometimes forcibly—and taken on long expeditions in remote wilderness (Harper, 2017), whereas in the latter, a close contact with the immediate social network is central and the interventions take place in nearby nature. As the American tradition originally aims at promoting the growth of the individual as a person, the individualistic approach and removing the individual from the family is understandable. On the other hand, in the friluftsterapi way and in SMOT, the focus is more explicitly on the relationship between the individuals and their surroundings in nature (Næss, 1990, p. 8–9). Carrying out the intervention close to home is, then, reasonable as this makes it in practice easier for the participating youth and families to adopt the friluftsterapi/SMOT practices into their everyday life. Further, working in multi-family groups encourages a sense of community: the families do not need to make it on their own; rather, they get to offer and receive support and connect with others who perhaps are in a similar life situation. We often explicitly encourage the families to keep in touch after the conclusion of the rehabilitation process, as a means to maintain connectedness over time.

Finally, on a global level, we have reached a devastating climax (IPCC, 2021) to where the relational approach urgently needs to be extended to include all living creatures of the more than human nature. Hendersson and Wamsler (2020) call for a new path toward a more conscious and connected society characterized by cooperation and interbeing instead of individualistic pursuit of profit and human dominance over nature. Being dialogical as a way of life is one way of conceptualizing this holistic relational approach (Seikkula, 2011) coupled with Næss’ (1990) understanding and practice of self-realization.

From an ethical perspective, increasing pro-environmental behavior through nature contact, and improving within- and between-family relationships, is the way toward achieving regenerative, self-sustaining communities. For parents and grandparents alike, there are few things as fulfilling in life as the ability to nurture, protect, and calm one’s own child or grandchild. Strengthening intra-familial connection and attachment may have a positive impact on future generations, local communities, and society in general. Through developing feasible, self-sustainable, and ethical outdoor healthcare practices, we seek to co-create a new path that leads family rehabilitation from the at times limiting indoor settings to exploring the possibilities that are to be discovered in nearby nature. We propose that healthcare services should adapt swiftly and radically to better respond to the complexity of families’ needs, where a recontextualization of individualistic, talk-based indoor psychological practices to systemic, holistic, and experiential family-focused interventions outdoors may foster connectedness in and to nature.

## Data Availability Statement

The raw data supporting the conclusions of this article will be made available by the authors, without undue reservation.

## Author Contributions

All authors contributed toward generating ideas and revised the final manuscript.

## Funding

The three developmental projects introduced in this contribution were financed by the Funding Centre for Social Welfare and Health Organizations (STEA).

## Conflict of Interest

The authors declare that the research was conducted in the absence of any commercial or financial relationships that could be construed as a potential conflict of interest.

## Publisher’s Note

All claims expressed in this article are solely those of the authors and do not necessarily represent those of their affiliated organizations, or those of the publisher, the editors and the reviewers. Any product that may be evaluated in this article, or claim that may be made by its manufacturer, is not guaranteed or endorsed by the publisher.
